# The Passing of the Ovary

**Published:** 1896-05

**Authors:** 


### THE PASSING OF THE OVARY.

"The times have changed," the ovary said;
"I am hopelessly out of date.
I have dropped from out the zenith of fame,
I have nothing left but a blasted name,
For Battey is dead, and Keith is dead,
And what has become of Tate?

My place in the alcohol jar is ta'en
By a blind, malicious worm.
It is hard for a lady of parts to be cut
By a mere cedilla under a gut!
But I'm out of the fashion and on the wane,
And you now triumphantly squirm.
So, Appendix, adieu,
It is time I withdrew—
*You may hear from me again.*"—

